# Age-Related Differences in Testosterone Concentration and Its Relation to Testicular Biometrics, Hemodynamics, and Fertility in Alpacas (*Vicugna pacos*)

**DOI:** 10.3390/vetsci10070429

**Published:** 2023-07-01

**Authors:** Manuel G. Pérez-Durand, Angela Massa-Guzmán, Natalio Luque-Mamani, Domingo A. Ruelas-Calloapaza, Jesús M. Urviola-Sánchez, Eloy A. Condori-Chuchi, Miguel A. Gutiérrez-Reinoso, Uri H. Perez-Guerra, Manuel García-Herreros

**Affiliations:** 1 Facultad de Medicina Veterinaria y Zootecnia, Universidad Nacional del Altiplano, Puno 21001, Peru; mgperez@unap.edu.pe (M.G.P.-D.); massaguzman@gmail.com (A.M.-G.); nluque@unap.edu.pe (N.L.-M.); druela@unap.edu.pe (D.A.R.-C.); jurviola@unap.edu.pe (J.M.U.-S.); 2 Escuela Profesional de Medicina Veterinaria, Laboratorio de Medicina de Animales Mayores, Univesidad Nacional San Antonio Abad del Cusco, Cusco 08000, Peru; eloy.condori@unsaac.edu.pe; 3 Carrera de Medicina Veterinaria, Facultad de Ciencias Agropecuarias y Recursos Naturales, Universidad Técnica de Cotopaxi (UTC), Latacunga 050150, Ecuador; miguel.gutierrez@utc.edu.ec; 4 Instituto Nacional de Investigação Agrária e Veterinária (INIAV), 2005-048 Santarém, Portugal

**Keywords:** alpacas, male, age-related fertility, testicular morphometry, testosterone, testicular hemodynamics, pregnancy rate

## Abstract

**Simple Summary:**

Studies related to alpaca (*Vicugna pacos*) reproduction are scarce in the Andean region. The potential age-related differences in serum testosterone profiles in alpaca males and its relation to testicular morphometric characteristics, hemodynamics, and pregnancy rate of alpaca females inseminated by natural mating was investigated as a model for the study of other South American camelid species. Under our experimental conditions, the results obtained from different age groups regarding testosterone concentration and ultrasonographic traits suggested that they were not determining factors for assessing potential fertility differences in alpaca males.

**Abstract:**

The goal of this study was to investigate the age-related differences in testosterone concentration and its relation to testicular biometrics, testicular blood flow, and fertility in alpacas (*Vicugna pacos*). Fifteen alpaca males with different ages (young (YM; ~12–14 mo.), n = 5; intermediate (IM; ~24 mo.), n = 5; and old (OM; ≥36 mo.), n = 5) were enrolled in the study. Blood samples were taken from each alpaca male and the circulating plasmatic testosterone concentration (TC; ng/mL) was determined using ELISA analysis. The testicular traits related to bio-morphometric parameters (the length (L), width (W), area (A), and volume (TV)) were assessed using B-mode ultrasonography. Pulse-wave/power Doppler ultrasonography was used to obtain the circulatory dynamic values (testicular hemodynamics) before the beginning of natural service mating. Significant differences were observed in TC among the age groups, increasing as the age of the males increased (2.47 ± 0.31, 8.45 ± 1.53, and 22.66 ± 2.15 for YM, IM, and OM, respectively; *p* < 0.05); however, no differences were observed regarding the testicular B-mode ultrasonographic parameters (L, W, and A) (*p* > 0.05). Positive correlations were observed between TV and testicular L, W, and A (r = 0.96, r = 0.95, and r = 0.96, respectively; *p* ≤ 0.001). Pulse-wave-Doppler-derived parameters such as the pulsatility index (PI) and the resistive index (RI), as well as the total vascularity area (TVA) assessed by power Doppler, were similar in all of the age groups studied (*p* > 0.05). General linear model (GLM) analysis showed a relationship between TC and TV (OR = 0.95; *p* = 0.04), as well as between TC and TVA (OR = 0.99; *p*= 0.02). Finally, no differences were observed regarding the pregnancy rate among the different age groups (*p* > 0.05). In conclusion, TC increased as the age of the alpaca males increased. Although TC was related to TV and TVA, the pregnancy rates obtained from individuals belonging to the different age groups were similar, indicating that TC, TV, and TVA were not determining factors in assessing the potential age-related fertility differences in alpaca males.

## 1. Introduction

South American camelid production is extremely important in the Andean region. Peru has a population of approximately 3.5 million of alpacas (*Vicugna pacos*), and 70% of the total population is owned by small- and medium-sized producers who carry out a traditional production system [1]. From a reproductive point of view, this management system implies the presence of between 6 and 10% of selected males within a flock, which reach puberty between 12 and14 months of age; however, the use of young males only starts to be efficient between the second and the third year of age, due to the existence of penis-preputial adhesion, which is a distinctive feature in camelids [2,3,4,5]. During the selection of breeding males, in addition to the evaluation of phenotypic characteristics, the male reproductive capacity is evaluated as well, which consists of determining the number of spermatozoa produced in relation to the testicular weight [6]. In other species, such as cattle, the estimation of sperm production is determined by the scrotal circumference [7,8]. Therefore, daily sperm production could be estimated externally by the testicular dimensions that determine the testicular volume [9]. 

Testicular dysfunction is a critical part of camelid breeding management programs. Sexual development plays a crucial role in many male reproductive processes, including those of camelid species. Age is considered a critical factor influencing the reproductive performance of camelids and other mammals. During development, the testicular tissue undergoes maturational processes such as changes in the intratesticular vascular endothelium morphology and the growth of Leydig cells. Sexual behavior, concentration of plasma testosterone, gametogenesis, testicular dimensions, and fertilizing potential are directly affected by the age of the male. The hypothalamic and gonadal hormonal interaction is linked to age and directly affects the plasma testosterone concentration. Testicular vascularity is the main pathway for hormone, oxygen, and nutrient distribution. Therefore, arterial blood supply is crucial for testicular metabolism and fertility, maintaining the pampiniform plexus below body temperature, which is essential for spermatogenesis.

Little attention has been paid to the hemodynamics and echotexture during the sexual development of South American camelids and its effects on pregnancy rate occurring during the peripubertal period and later periods. The reduction in reproductive potential may be attributable to age, which could negatively affect the testicular blood perfusion, testosterone production, oxygen supply, and fertility. However, many factors related to the control of testicular microcirculation are still unknown. Therefore, in order to evaluate the inside of the reproductive organs, advanced ultrasonographic techniques that can detect abnormalities, parenchyma, and testicular morphometry are needed. In addition, ultrasonography has been characterized as a non-invasive technique and generally does not require the use of general anesthesia or sedation [10,11].

Conventional gray-scale ultrasonography (B-mode) has been routinely used for testicular parenchyma evaluation, due to the fact that echogenicity is related to the histomorphology of the seminiferous tubules but lacks information about the organ vasculature [12]. Non-invasive imaging technologies, such as color and pulse-wave Doppler ultrasonography, can be used as alternative diagnostic techniques for evaluating structural and functional hemodynamics (the fluctuation of testicular microcirculatory blood flow) in order to determine the testicular function, and, therefore, male fertility [13]. The pulsatility index and the resistive index are indices that are obtained from intratesticular blood flow velocity (peak systolic velocity and end-diastolic velocity) and have been described as indicators of male fertility in several species. The increased testicular blood flow of arterial microvessels can result in increased fertility because of its positive impact on spermatogenesis. Power Doppler, on the other hand, measures the number of cells passing through the piezoelectric crystals in a unit of time and is, therefore, much more commonly used in areas with slow blood flow [14,15]. However, color, pulse-wave, and power Doppler ultrasonography have not often been used in camelids. All of these technologies could allow for a better understanding of testicular physiology in male alpacas, as well as being alternative methods for the evaluation of their reproductive capacity.

To our knowledge, there are no studies on the effects of age on testicular morphology, hemodynamics, testosterone level profiles, or fertility in terms of pregnancy rate in alpacas. For this reason, the aim of the present study was to study the potential age-related differences in serum testosterone profiles in alpaca males and its relation to the testicular morphometric characteristics, hemodynamics, and pregnancy rate of alpaca females inseminated by natural mating.

## 2. Materials and Methods

### 2.1. Ethical Statement

This study was conducted according to the guidelines of the Declaration of Helsinki and followed the Code of Ethics for animal experiments, as reflected in the ARRIVE guidelines, which are available at: http://www.nc3rs.org.uk/ARRIVEchecklist (accessed on 1 June 2022). This study was approved by the Bioethics Committee for the use of experimental animals at the Universidad Nacional del Altiplano, Puno, Peru (approval date: 1 January 2019, code number: DE-000399-2019).

### 2.2. Reagents and Media

Unless otherwise stated, all reagents and materials were purchased from Sigma (Sigma-Aldrich, St. Louis, MO, USA).

### 2.3. Animals and Management

Fifteen Suri breed fertile alpaca males (age: 12 to 40 mo.; weight: 45–60 kg) were used in the present study. They were clinically healthy individuals and free from any reproductive or cardiovascular issues. The alpacas were kept in an outdoor paddock at the Chuquibambilla Experimental Research Centre, District of Umachiri, in the Peruvian Andean highlands (~4000 m.a.s.l.; latitude: 14°47′16.46″ S; longitude: 70°43′42.57″ W) exposed to natural environmental conditions. The animals were allocated into three experimental groups (n = 5 each). The experimental male groups were composed of the following: (a) ~12–14 mo. (young individuals), (b) ~24 mo. (intermediate individuals), and, finally, (c) ≥36 mo. males (old individuals). The age groups were selected based on the Experimental Research Centre’s reproductive management scheme, where males that are 36–40 mo. old (38 ± 2 mo.) are retired from the flock production system to avoid inbreeding-derived issues. For the current study, only sexually mature fertile alpaca males without penile-preputial adhesions were selected, irrespective of the age group (including young individuals). All groups were maintained under the same nutritional (natural pasture) and management conditions.

### 2.4. Blood Sampling and Hormone Analysis

Before every single ultrasound assessment session (once per week, total n = 4 per male), blood samples (one per session, total n = 4 per male) were taken early in the morning (7:00 a.m.) from each alpaca male by jugular venipuncture after disinfection using Idogel^®^ (70% alcohol gel, Lab. Roker, Perú) into sterile vacutainer tubes (BD Vacutainer; BD, USA; 5 mL) containing EDTA. All samples were centrifuged for 15 min at 2500× *g*. at 4 °C. Plasma was collected and stored at −20 °C for further hormonal analysis. The circulating plasmatic testosterone concentrations (ng/mL) were determined using a commercial ELISA kit (Testosterone ELISA Kit, Abcam, MA, USA) according to the manufacturer’s specifications. The intra- and inter-assay coefficients of variation were 3.3 and 4.8%, respectively, and the assay sensitivity was 0.05 ng/mL. The results were obtained using an ELISA plate reader (Organon Teknica, Microwell System, model reader 230S, China) according to the recommendations from Perez-Guerra et al. [16] for camelids.

### 2.5. Testicular Analysis Using B-Mode Ultrasonography

The testicular traits (length (L), width (W), and area (A)) were assessed using B-mode ultrasonography using an ultrasound machine (US Draminski 4Vet^®^, Draminski ul. Owocowa, Olsztyn, Poland) equipped with a 9.0 MHz linear transducer. All machine settings, such as brightness, frequency, depth, and contrast, were standardized and maintained during all ultrasonographic assessments. Regarding the testicular ultrasound observations, hypo-echoic texture (background) and homogenous echogenicity (medium) were observed in the testicular parenchyma. Moreover, hyper-echogenic peripheral capsules (tunica albuginea; parietal and visceral tunics) and a hyper-echoic central line (mediastinum testis) were detected. Longitudinal and transversal testicular measurements were evaluated by ultrasonography. The testicular volume was calculated using the following ellipsoid formula: (length (L), width (W), and height (H) × 0.5236)) [17].

### 2.6. Testicular Assessment Using Doppler Ultrasonography

The alpaca males were examined for changes in testicular hemodynamics. To minimize effects on testicular blood flow, the animals were physically restrained without sedation. All ultrasonographic scanning procedures were carried out by the same technician using an ultrasound scanner, known as US Draminski 4Vet^®^ (Draminski ul. Owocowa, Poland), equipped with a linear transducer (9.0 MHz). The alpacas were scanned in the standing position. Before each scanning procedure, wool was shaved from the scrotum area and gel was applied to reduce imaging artefacts. The equipment settings were standardized (frequency: 9.0 MHz, brightness: moderate, depth: 5 cm, contrast: uniform, wall filter: 5 cm/s, and sample gate: 2.0 mm). For the pulse-wave Doppler assessment, the gate was set at 0.5 mm, the angle between the Doppler beam and the long axis of each vessel was ~60°, and the high-pass filter 50 Hz, which were maintained throughout the experimental period. A minimum of three consecutive waves were analyzed. The transducer was positioned vertically on the lateral surface of the testes until the supra-testicular artery, located cranio-ventral to the head of the epididymis to the vascular network, including marginal and intratesticular arteries, were visible, as recommended by [18] in llamas. The following blood flow parameters were estimated four times (once per week for four weeks in a row): resistive index (RI: (PSV-EDV)/PSV)), pulsatility index (PI: (PSV-EDV)/mean velocity), and colored area toward the testes/pixels for pampiniform plexus vascularization assessment. The pampiniform-colored area/pixels divided by the area of the region/pixels was used to calculate the percentage of colored areas that represented the blood vessels, and the corresponding flows were red (blood flowing towards the transducer) and blue (blood flowing away from the transducer) in the right and left testes. To determine the testicular vascular area (TVA; %) the same ultrasonographic equipment was used, activating the Doppler power mode. The gray-scale digital images of the testicular parenchyma were analyzed, and testicular echogenicity (pixel intensity) was evaluated using non-overlapping rectangular regions (~1 cm^2^). Each testicle was identified separately for each animal, and the images were saved in short videos. Subsequently, four images were obtained and analyzed from each video using Image J^®^ software (Java-based image processing, Laboratory for Optical and Computational Instrumentation (LOCI, Madison, WI, USA), which allowed us to determine the TVA parameter, similar to that reported for obtaining the luteal vascular area [19,20]. The testicular area was obtained using the following formula: TVA (%) = (testicular vascular area)/(testicular total area) × 100.

### 2.7. Male Fertility Assessment by Evaluation of Pregnancy Rate

The male fertility potential in the different experimental groups was determined by using an average of 30 females (age: 12 to 60 mo.) per male during the whole reproductive season based on the Experimental Research Centre’s reproductive management scheme (from January to March, approximately 3 months). All males were previously separated per age group. Every mating day, all males were separated individually (one male per pen) to avoid any interference among them. The sexually receptive females were grouped by the technical staff after daily estrus detection and the controlled mating process was carried out. The males and females were randomly chosen to carry out the mating. The mating process between the males and females was carried out in separated pens and lasted between 10 and 15 min [9,21,22]. The pregnancy rate was analyzed after 35 days post-mating by transrectal ultrasonographic evaluation using US Draminski 4Vet^®^ equipment (Draminski ul, Owocowa 17, Olsztyn, Poland) with a linear transducer (frequency: 9.0 MHz; depth: 6 cm). The females were determined to be pregnant when the existence of a gestational sac and the echogenic presence of the embryo were confirmed [23,24].

### 2.8. Statistical Analysis

The data matrix was evaluated using descriptive statistics to determine measures of central tendency, dispersion, normality assumptions, and homoscedasticity. Subsequently, the experimental groups were subjected to a one-way ANOVA, and, then, they were compared using Tukey’s post-hoc test to determine differences among the means. All of the variables were analyzed to test their relationship with testosterone concentration using the generalized linear model (GLM). Finally, the Pearson’s correlation test was carried out to determine the degree of association between the variables. All analyses were performed using R-Studio software [25].

## 3. Results

### 3.1. Age-Related Effects on Testosterone Concentration, Testicular Morphometric Parameters, and Pregnancy Rate in Alpacas

Table 1 shows the testosterone concentration, testicular morphometric parameters, and pregnancy rate among the three experimental age groups in alpacas. Significant differences were observed in the testosterone concentrations among the age groups, increasing as the age of the males increased (*p* < 0.05); however, no differences were observed regarding the testicular morphometric parameters (length, width, area, and volume) or pregnancy rate among the different groups (*p* > 0.05; Table 1).

### 3.2. Age-Related Effects on Testicular Circulatory Dynamics in Alpacas

**Figure 1:**
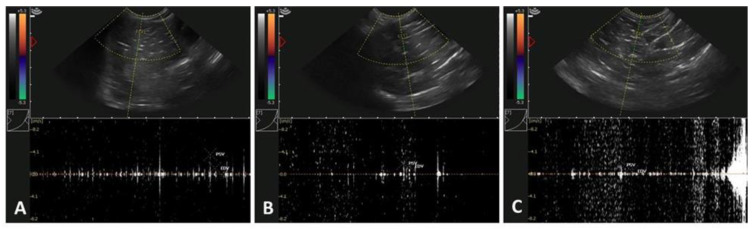
Evaluation of testicular parameters (resistive index (RI) and pulsatility index (PI)) by using Doppler (pulse-wave) ultrasonography in male alpacas in the three experimental age groups. Representative ultrasonographic pulse-wave Doppler images obtained from testicular tissue in alpacas (*Vicugna pacos*): (**A**) young males (age: ~12–14 mo.); (**B**) intermediate males (age: ~24 mo.); and (**C**) old males (age: ≥36 mo.).

Figure 1 and Table 2 show the Doppler (pulse-wave) ultrasonographic characteristics of the different age groups of male alpacas.

Although no significant differences were observed in any of the variables studied (RI, PI, and TVA), irrespective of the age groups analyzed, the values were slightly greater in the young and old males (*p* > 0.05; Table 2).

**Figure 2:**
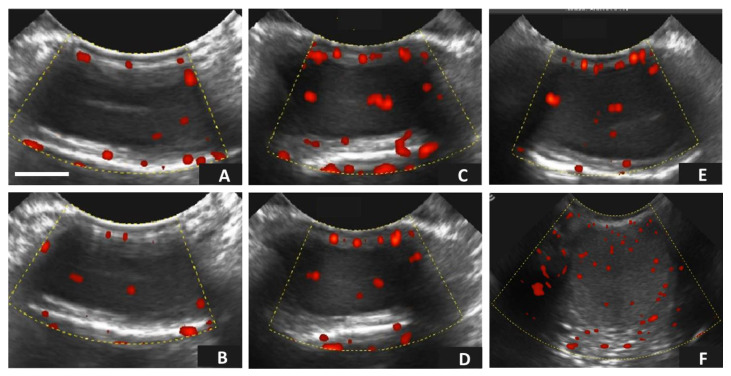
Assessment of testicular hemodynamics [(red dots: testicular vascular area (TVA)] by using Doppler (power) ultrasonography in male alpacas in the three experimental age groups. Representative ultrasonographic power Doppler images obtained from testicular tissue in alpacas (*Vicugna pacos*): (**A**,**B**) Testicular hemodynamics (TVA) obtained from young males (age: ~12–14 mo.) in the right (**A**) and left (**B**) testicles, respectively; (**C**,**D**) Hemodynamic patterns (TVA) obtained from intermediate males (age: ~24 mo.) in the right (**C**) and left (**D**) testicles, respectively; and (**E**,**F**) Testicular hemodynamics (TVA) obtained from old males (age: >36 mo.) in the right (**E**) and left (**F**) testicles, respectively. Scale bar: 10 mm.

Figure 2 shows the Doppler (power) ultrasonographic characteristics of the different age groups of male alpacas. No statistical differences were observed among the different groups (*p* > 0.05). 

### 3.3. Testosterone Concentration and Its Relation to Testicular Morphometry, Hemodynamics, and Pregnancy Rate in Alpacas

Table 3 shows the relationship, analyzed by GLM, between the independent ultrasonographic variables obtained from the B-mode and Doppler images and the pregnancy rate, with respect to the dependent variable of testosterone concentration. A significant relationship was observed between the testicular volume and TVA, with respect to testosterone concentration (*p* < 0.05); however, no relationship was observed between the rest of the ultrasonographic variables and pregnancy rate with regard to the testosterone concentration (*p* > 0.05).

### 3.4. Correlation between Testosterone Concentration, Testicular Morphometry, Testicular Hemodynamics, and Pregnancy Rate in Alpacas

Table 4 shows the Pearson’s correlation coefficients between the morphometric variables obtained by B-mode ultrasonography, the hemodynamic variables obtained by Doppler ultrasonography, testosterone concentration, and pregnancy rate in alpacas. Strong and positive correlations were observed between all of the testicular lengths, widths, areas, and volume parameters obtained from the B-mode parameters (*p* < 0.001). Moreover, significant positive correlations were detected between all of the morphometric parameters obtained from the B-mode and the Doppler-derived ultrasonographic parameters PI (*p* < 0.05). No significant correlations were observed between the rest of the morphometric, hemodynamic, hormonal, or pregnancy rate parameters (*p* > 0.05; Table 4).

## 4. Discussion

To the best of our knowledge, this is the first study to investigate the effects of age on the testicular morphometry, blood flow, testosterone concentration, and pregnancy rate of alpacas. There was evidence to suggest that the testicular blood flow increased with the age of the animals; however, no differences were observed regarding the testicular volume among the different age groups. On the other hand, marked differences were detected with regard to the testosterone concentration, which increased with the age of the animals, being 4-fold greater in the medium age group compared to the young males and 3-fold greater when the intermediate age group and the old males were compared. Reports on the study of reproductive physiology and hormone profiles in camelids are limited; however, some studies have reported that testosterone production reached the highest level at 24 months of age, coinciding with the onset of puberty, which was related to the testicular Leydig cells population, which increases in number from 9 months of age onwards [2,26,27]. The results obtained in the present study showed that the increase in testosterone concentration was related to the age of the alpacas. In the present study, the testosterone concentrations were different in all of the age groups, with the lowest concentration being observed in the younger males (~12–14 months), followed by the intermediate-age males (~24 months), and, finally, the highest concentration was observed in the older males (≥36 months). This same behavior, in relation to testosterone levels and more advanced sexual maturity, has been reported by several authors, whose results were similar to those observed in the present study [28,29].

The measurement of B-mode ultrasonographic testicular characteristics is a more reliable tool for the selection of breeders compared to classical measurements such as scrotal circumference or the use of a Vernier ruler [30,31]. Ultrasonographic measurements more accurately define the testicular tissue that is characterized by homogeneity and medium echogenicity by disregarding other structures such as skin, subcutaneous tissue, testicular fascia, and epididymis that could influence the accuracy of the testicular measurements [32,33]. In the present study, the B-mode ultrasonographic characteristics were similar in the three age groups, indicating that ~12–14 mo. old males could already be considered as fertile breeders, taking into account the measurements of testicular length, width, area, and volume. Moreover, the values obtained for the testicular length and width were similar in all three age groups. These characteristics were similar to what has been previously reported in adult vicuñas aged between 3 and 10 years [34], and also in alpacas, in which testicular measurements were evaluated by ultrasonography [32]. Moreover, similar testicular length and width measurements have been described in alpacas between 12 and 113 months of age, and were similar to those observed in the present study [29,35]. The testicular area and volume were similar in all three groups, indicating that all of the males were sexually mature from the point of view of testicular morphometry measurements, whose testicular dimensions reached definitive measurements at 12–14 months of age, when alpacas reach sexual puberty [36].

Pulsed Doppler parameters, such as PI and RI, are characterized by the determination of blood flow in organs [37]. Both indices are independent of the angle of evaluation (as is the case with color Doppler) [18]. In the case of the testis, the RI measures the blood flow determined by the resistance of the testicular vascular network, while PI quantifies the pulsatility, or oscillations, in the form of waves produced by the blood circulation at the testicular level [38]. In canines, studies have shown an inverse relationship between both of these indices and sperm motility [39]. Therefore, it can be assumed that the lower both of these indices are, the more efficient spermatogenesis will be [40,41,42]. The PI and RI indices reported in the present study were apparently normal for breeding males in alpacas under altitude conditions [15]. On the other hand, the PI and RI measurements were higher in the older males compared to the other groups. This difference was probably due to the degenerative changes that increase testicular tissue resistance in older males, as has been reported in other species, such as equines [40]. The PI and RI were not different when the three age groups were compared. Thus, the resistance indices obtained were slightly lower compared to those reported by other authors, who evaluated fertile and infertile llamas [18]. However, in the present study, both of the indices were higher in the males belonging to the young and old male groups compared to the middle-aged male group. This fact has also been observed in a similar study in sheep, with higher results in the young and old male groups, but not in the middle-aged males [43]. The Doppler characteristics in male alpaca breeders had a range of RI between 0.49 and 0.57, a PI between 0.31 and 0.53, and a TVA between 3.77% and 9.16%, which could be considered as apparently normal in altitude conditions because the animals evaluated were proven fertile males that were tested during several breeding seasons.

Regarding the relationship between the serum testosterone levels and the morphometric characteristics obtained by B-mode parameters, there were differences only in the testicular volume parameter and testicular vascular area in relation to higher or lower testosterone concentrations. On the other hand, the other morphometric variables were not significantly different. The testosterone concentration of males has been significantly related to TV and TVA in other species [30,44]. However, there is controversy regarding the testicular volume, specifically related to the relationship between testicular volume for predicting testosterone levels in humans and camelids, reporting high sensitivity and specificity in the analyses performed [28,45]. On the other hand, testicular size varies with age, and there is also an increase in testicular volume that corresponds to an increase in the amount of serum testosterone in camelids [46]. Likewise, there is a relationship between the testicular volume and the amount of Leydig cells (the cells in charge of testosterone production), which varies according to the histological pattern [47]. However, some authors report that there is no relationship between testicular morphometric parameters and testosterone production [29]. The relationship between testosterone concentration and TVA in the present study may be due to the fact that the testes are compact organs that need constant blood flow [46]. Several authors have stated that blood flow may be a marker to predict testicular functions in males because, anatomically, the testicular artery is branched and has an important endocrine function in hormone secretion (steroidogenesis) and exocrine function related to sperm production (spermatogenesis) [42,43,48,49].

Regarding the relationship between the B-mode ultrasonographic variables, strong positive correlations were observed between the morphometric parameters analyzed. The PI Doppler parameter showed intermediate correlations with the morphometric parameters of testicular volume, area, width, and length. The TVA parameter showed a low correlation with the morphometric parameters of testicular length, width, area, and volume. Finally, the RI parameter showed intermediate negative correlations with the testicular volume, area, width, and length. When testosterone levels and B-mode ultrasonographic characteristics were analyzed, the correlation coefficients observed were close to zero, indicating that there was no marked relationship between the testosterone concentration and the morphometric parameters. However, other authors have reported no correlations between testicular width and length and testosterone levels [29]. The strong positive correlations observed could be due to the fact that the B-mode morphometric characteristics were related to each other, as mentioned in other studies in camelids and other species [28,29,33]. On the other hand, the RI Doppler parameter was negatively correlated with the other parameters, which is apparently normal due to the low arterial resistance of the testes, increased testicular perfusion, nutrient availability, and oxygenation [42]. On the other hand, it could also be related to increased exocrine and endocrine functionality [14]. However, in the present study, the PI parameter showed an intermediate positive correlation with respect to the morphometric parameters obtained by B-mode ultrasonography, and, therefore, it may be necessary to further evaluate this phenomenon in subsequent studies.

The studies on the relationship between B-mode parameters, Doppler, testosterone, and pregnancy rate in camelids are lacking. Most of the existing studies are descriptive or only characterize fertile and infertile alpacas [18,29]. This is the first study on the relationship between different hormonal and ultrasonographic variables and pregnancy rate in alpacas. In canines, negative correlation coefficients were observed when sperm motility was compared to PI and RI indices [50]. In the present study, similar results were observed in alpacas, showing a negative correlation between PI and RI indices and pregnancy rate. Furthermore, in the present study, male alpacas of different ages were used and a negative correlation between pregnancy rate and RI was observed. Several studies of humans and canines have demonstrated that an increase in RI was associated with testicular problems, especially when the RI was higher than 0.6 [50,51]. However, in the present study, despite observing a negative correlation between RI and pregnancy rate, no differences were observed between the different age groups. It should be noted that the fertilization process and subsequent pregnancy could be related to other intrinsic and extrinsic factors derived from the males, as well as the females, which could explain the negative correlations obtained between the pregnancy rate and the hormonal and ultrasonographic parameters studied. Finally, the B-mode parameters, Doppler, and testosterone concentration showed negative correlation coefficients in relation to fertility. It is important to note that the ultrasonographic parameter PI and the hormonal parameter testosterone concentration had a correlation coefficient close to zero, therefore, their relationship with the pregnancy rate can be determined as non-existent.

South American camelids show a marked reproductive seasonality in the Andean region. Mating within 15 to 20 days after parturition improves fertility rates [6,52]. In the present study, all of the females were managed in the same way and the mating was carried out following a controlled procedure. As a consequence, no differences between the groups were detected. South American camelids have a high rate of embryonic loss during the first 60 days of pregnancy [53]; however, the factors contributing to such high embryonic mortality rates are still unknown. The pregnancy rate could be associated with the fertility rate of the male alpacas, as they may show significant differences during mating [54]. In the present study, no differences in pregnancy rate were observed between females mated by males from different age groups. In general, embryo implantation is not only dependent on male fertility, but also involves extensive remodeling of the uterine epithelial surface [55] and of the extracellular matrix (ECM) within the endometrium, in which collagen and matrix metalloproteinases (MMPs) play essential roles [55]. Other factors associated with infertility in alpacas and llamas are related to the presence of persistent hymen [56]; however, in the present study there was no incidence of this factor. On the other hand, old (>15 years) and low-body-condition-score (<2) female alpacas may influence pregnancy rates [57]. However, in the present study, the females used were younger and their body condition score was never <2. These factors may have resulted in no significant differences in pregnancy rates between the groups, indicating that there were no influential factors related to the females used.

Finally, the fact that the pregnancy rates were similar among the male groups could be explained by the fact that, in the present study, only sexually mature fertile alpaca males without penile-preputial adhesions were selected, irrespective of age (including young individuals). It is important to note that, in the present study, there was a previous young male selection depending on the matting potential (this included a previous testicular ultrasonographic assessment). According to Abraham et al. [35], the use of testicular morphometry (specifically the length of the testicles) was better than age alone to predict sperm production, explaining the individual onset of puberty, and is a better guide for reproductive management recommendations. Therefore, alpaca males are also selected for breeding selection based on testis size. In addition, according to Sumar [58], at 1 year of age, the males show sexual interest in the females, and around 10% of alpaca males have complete liberation of penis-prepuce adhesions and are capable of performing copulation. Finally, according to Tibary et al. [59], the age at puberty varies (from 1 to 3 years old) depending on, among other factors, genetics, nutritional status, climate, and at what time of year the individual was born. Precocious behavior and early mating are considered to be desirable traits in genetic selection programs.

## 5. Conclusions

In conclusion, the B-mode (bio-morphometric parameters) and Doppler (RI, PI, and TVA) ultrasonographic characteristics evaluated were similar in the three age groups of alpaca males. The testosterone concentration was related to the volume and percentage of the testicular vascular area. Moreover, differential correlations were also observed among the variables studied. The pregnancy rates obtained from individuals belonging to the three age groups were similar, indicating that age, testosterone concentration, and testicular vascular area were not determining factors for assessing potential age-related fertility differences in alpaca males.

## Figures and Tables

**Table 1:** Testosterone concentration, ultrasonographic (B-mode) morphometric characteristics, and pregnancy rate obtained from the different age groups of male alpacas.

Experimental Groups	n	Testosterone (ng/mL)	Testicular Length (mm)	Testicular Width (mm)	Testicular Area (cm^2^)	Testicular Volume(cm^3^)	Pregnancy Rate (%)
YM (~12–14 mo.)	5	2.47 ± 0.31 ^a^	36.75 ± 3.05 ^a^	21.59 ± 1.91 ^a^	6.59 ± 1.09 ^a^	11.69 ± 2.69 ^a^	86.85 ± 3.06 ^a^
IM (~24 mo.)	5	8.45 ± 1.53 ^b^	34.1 ± 1.30 ^a^	19.69 ± 0.65 ^a^	5.14 ± 0.50 ^a^	8.77 ± 1.24 ^a^	89.06 ± 2.44 ^a^
OM (≥36 mo.)	5	22.66 ± 2.15 ^c^	38.01 ± 1.55 ^a^	21.01 ± 1.64 ^a^	6.48 ± 0.69 ^a^	10.93 ± 1.43 ^a^	84.01 ± 3.06 ^a^

Values are expressed as mean ± S.E.M. The different letters within the same column (^a–c^) represent statistical differences among the groups (*p* ≤ 0.05). YM: young males; IM: intermediate males, and OM: old males.

**Table 2:** Ultrasonographic parameters (pulse-wave/power Doppler) in male alpacas (*Vicugna pacos*) of different ages.

Experimental Groups	n	Resistive Index (RI)	Pulsatility Index (PI)	Test. Vascular Area (% TVA)
YM (~12–14 mo.)	5	0.54 ± 0.03	0.53 ± 0.08	9.16 ± 4.83
IM (~24 mo.)	5	0.49 ± 0.06	0.31 ± 0.09	3.77 ± 0.75
OM (≥36 mo.)	5	0.57 ± 0.05	0.47 ± 013	3.84 ± 0.25

Values are expressed as mean ± S.E.M. No statistical differences were observed among the different groups (*p* > 0.05). YM: young males; IM: intermediate males, and OM: old males.

**Table 3:** Generalized linear model (GLM) analysis showing the relation between the testosterone concentration and the ultrasonographic variables (B-mode and Doppler) and fertility in alpacas (*Vicugna pacos*).

Variable	Β	Standard Error	Exp (B)/ (OR)	Z	*p*
Intercept	0.24	0.01	1.27	16.29	<0.0001 *
Testicular Length	0.02	0.01	1.02	1.93	0.102
Testicular Width	−0.02	0.02	0.98	−0.89	0.410
Testicular Area	0.09	0.05	1.09	1.74	0.133
Testicular Volume	−0.05	0.02	0.95	−2.36	0.046 *
Pregnancy Rate	0.05	0.31	1.18	0.18	0.860
Resistive Index	−0.22	0.18	0.8	−1.23	0.266
Pulsatility Index	0.02	0.09	1.02	0.18	0.863
Test. Vascular Area	−0.01	0.00	0.99	−2.90	0.027 *

Testicular length (mm); testicular width (mm); testicular area (cm^2^); testicular volume (cm^3^); pregnancy rate (%); resistive index (-); pulsatility index (-); test. Vascular area (%). B represents the unstandardized regression weight (multiple linear regression weight); standard error represents the standard deviation of the coefficient point estimate in the GLM; Exp (B)/(OR: odds ratio) represents the predicted change in odds for a unit increase in the predictor; Z represents the statistic for testing the hypothesis that the corresponding parameter (regression coefficient) is zero. *: *p* ≤ 0.05.

**Table 4:** Pearson’s (r) correlation indices between the different ultrasonographic (B-mode and Doppler) parameters, testosterone levels, and pregnancy rate in alpacas (*Vicugna pacos*).

	Testosterone	Testicular Length	Testicular Width	Testicular Area	Testicular Volume	Resistive Index	Pulsatility Index	Testicular Vascular Area	Pregnancy Rate
Testosterone	-								
Testicular Length	0.20	-							
Testicular Width	0.07	0.90 ***	-						
Testicular Area	0.07	0.91 ***	0.97 ***	-					
TesticularVolume	0.08	0.96 ***	0.95 ***	0.96 ***	-				
Resistive Index	−0.01	−0.30	−0.33	−0.33	−0.44	-			
Pulsatility Index	0.02	0.68 *	0.59 *	0.61 *	0.66 *	−0.10	-		
Test. VascularArea	−0.52	0.25	0.25	0.40	0.27	−0.08	0.26	-	
Pregnancy rate	0.03	−0.45	−0.54	−0.48	−0.37	−0.18	0.01	−0.15	-

Significance between parameters is shown as * (*p* ≤ 0.05) and *** (*p* ≤ 0.001).

## Data Availability

All data generated or analyzed during this study are included in this article.
